# Beyond the Usual Suspects: IgG4-Related Disease as a Rare Culprit in Cardiac Valvular Disorders

**DOI:** 10.3390/life16040537

**Published:** 2026-03-24

**Authors:** Piera Costanzo, Savino Sciascia, Giacomo Quattrocchio, Pierluigi Sbarra, Antonella Barreca, Roberta Bracci, Irene Cecchi, Massimo Radin, Elisa Menegatti, Simone Baldovino

**Affiliations:** 1 Department of Clinical and Biological Sciences, University of Turin, 10125 Turin, Italy; piera.costanzo@unito.it (P.C.); savino.sciascia@unito.it (S.S.); irene.cecchi@unito.it (I.C.); massimo.radin@unito.it (M.R.); elisa.menegatti@unito.it (E.M.); 2 SC of Cardiology, San Giovanni Bosco Hospital, ASL Città di Torino, 10154 Turin, Italy; 3 University Center of Excellence on Nephrologic, Rheumatologic and Rare Diseases (ERK-Net, ERN-Reconnect and RITA-ERN Member) with Nephrology and Dialysis Unit and Center of Immuno-Rheumatology and Rare Diseases (CMID), Coordinating Center of the Interregional Network for Rare Diseases of Piedmont and Aosta Valley, ASL Città di Torino, San Giovanni Bosco Hub Hospital, 10154 Turin, Italy; 4 Division of Pathology, Azienda Ospedaliero-Universitaria Città della Salute e della Scienza, Department of Medical Sciences, University of Turin, 10126 Turin, Italy; antonella.barreca@unito.it; 5 SC Transplant Coordinating Service, Azienda Ospedaliero-Universitaria Città della Salute e della Scienza, 10126 Turin, Italy

**Keywords:** IgG4-related disease, valvular heart disease, autoimmune cardiopathy, cardio-rheumatology, rare cardiac diseases

## Abstract

Cardiologists consider degenerative or infectious causes when evaluating valvular heart disease. However, the role of autoimmune disorders, though less frequent, remains clinically significant. This report describes a young male patient presenting with persistent coronary disease and a suspected valvular cusp perforation initially attributed to infective endocarditis, which ultimately proved to be a manifestation of IgG4-related disease. IgG4-related disease is a rare condition, more prevalent in Asian populations, that typically affects the pancreas, salivary glands, lacrimal glands, and the retroperitoneum. Cardiac involvement, although uncommon, can present in various ways, including pericarditis, pulmonary arterial hypertension, valve dysfunction, cardiac pseudotumor, and coronary disease. Diagnosing and managing IgG4-related cardiac involvement requires heightened clinical suspicion, serological and histopathological assessment, and prompt interdisciplinary collaboration. Notably, involving rheumatologists in the management of these rare yet impactful autoimmune cardiac diseases is essential.

## 1. Keypoints

1. Autoimmune diseases can also cause valvular heart disease.
2. IgG4-related disease is an uncommon autoimmune disorder that affects multiple organs, including the heart, and is more prevalent in Asians.
3. Cardiac manifestations of IgG4-related disease are diverse, including valve dysfunction and coronary disease.
4. Management requires high clinical vigilance and collaboration between cardiologists and rheumatologists.

## 2. Introduction

In the modern clinical setting, cardiovascular diseases are mainly regarded as degenerative processes or infectious causes. However, a significant yet often under-recognised segment of cardiac pathology arises from autoimmune and systemic inflammatory disorders [1].

Among these, Immunoglobulin G4-related disease (IgG4-RD) has emerged as a complex, multi-organ fibro-inflammatory condition that presents a significant diagnostic challenge for clinicians. First formally described in 2001, this syndrome is characterised by tissue infiltration of IgG4-positive plasma cells, storiform fibrosis, and often, elevated serum IgG4 levels. While it frequently involves the pancreas and salivary glands, its cardiovascular manifestations—including aortitis, periaortitis, and coronary periarteritis—are increasingly recognised as critical, life-threatening components of the disease spectrum [2].

The epidemiology of the disease suggests a prevalence of approximately 1 to 4.6 per 100,000, primarily affecting males aged 50 to 70, although younger cases are also documented [3].

For cardiologists, IgG4-RD is particularly difficult to diagnose because it can mimic more common conditions. It may appear as aggressive, relapsing coronary artery disease (CAD) that resists standard revascularisation techniques, or as valvular lesions that closely resemble infective endocarditis. The diagnosis can be delayed for many years, sometimes up to 13 years, during which irreversible organ damage or sudden cardiac death may occur [4].

This paper discusses the case of a 40-year-old man with a ten-year-long medical history of recurrent myocardial infarctions and valvular dysfunction, which was ultimately attributed to IgG4-RD. By analysing this case, we aim to provide essential insights and diagnostic advice for cardiologists, highlighting the importance of looking beyond conventional risk factors when encountering atypical or resistant cardiac cases to enable timely intervention with targeted immunosuppression.

## 3. Case Description

A 40-year-old man came to our attention in 2013 for acute anterior myocardial infarction, for which he underwent urgent coronary revascularisation on the anterior descending (LAD) and circumflex (LCx) with stent placement. He presented a positive family history of cardiovascular disease and dyslipidemia and was a previous smoker.

However, due to his young age, we investigated his medical history to find some less common risk factors for CAD. In 2010, he had a diagnosis of autoimmune thyroiditis and chronic discoid lupus of the ear—this diagnosis was biopsy-proven, with a histological finding of infiltration of perivascular spaces by lymphocytic elements and IgM- and C3-positive immunofluorescence with a speckled pattern on the basal membrane; serological testing for systemic autoimmunity performed at that time showed 1:80 Antinuclear Antibodies (ANA) with a fine, speckled pattern at immunofluorescence on Hep2 cells. First, he was treated with low-dose steroids orally and hydroxychloroquine 200 mg a day, and subsequently only with hydroxychloroquine.

During his hospitalization in 2013, we performed a complete autoantibody screening for Systemic Lupus Erythematosus (SLE)—i.e., ANA, anti-extractable nuclear antigens (ENA) antibodies, anti-double-stranded DNA (dsDNA) antibodies, research for Lupus Anticoagulant (LAC) and Antiphospholipid antibodies (APLs), including anti Beta 2 GP1, and anticardiolipin IgG and IgM antibodies. All the tests performed were negative upon admission and after 12 weeks.

In November 2014, the patient was readmitted for unstable angina and episodes of paroxysmal atrial fibrillation, for which he was treated with warfarin. The coronary angiography showed in-stent restenosis on LAD and LCx and disease progression on the first diagonal and intermediate branches. So he underwent surgical coronary revascularization with the left internal mammary on LAD and diagonal branch, and right internal mammary on CFX and intermediate branch.

Before the surgical intervention, the patient was evaluated by a rheumatologist consultant, concluding with a discoid LE diagnosis without any sign of SLE.

In 2016, he had a new episode of angina, and coronary angiography showed a chronic total occlusion of the right coronary artery that was treated with angioplasty and two drug-eluting stents (DES).

In 2017, considering the presence of multiple symptomatic episodes of atrial fibrillation and the young age of the patient, a pulmonary vein ablation procedure was performed, and treatment with the direct oral anticoagulant rivaroxaban was started.

In 2018, he was hospitalized again for an episode of angina during an atrial flutter paroxysm; the coronary angiography showed chronic coronary restenosis on the middle right coronary and diffuse progression of disease on native branches with total coronary occlusion of the first marginal branch, treated with angioplasty and DES placement. The ejection fraction slightly reduced from 45% in 2013 to 40% in 2018.

In May 2021, he completed the SARS-CoV2 RNA vaccine cycle without complications. Hydroxychloroquine was interrupted a few months before due to complete remission of discoid LE.

However, he kept complaining of fatigue, dyspnea, and cardiac palpitations due to recurrent atrial flutter paroxysms, for which he was already on a waiting list for a new attempt at atrial flutter ablation.

In August 2021, at 48, he returned with syncope, acute heart failure, atrial flutter at a heart rate of 300 bpm, and pneumonia with slight hyperthermia. He tested negative for SARS-CoV-2. HRCT showed limited pneumonia in the left superior lobe, possible bibasal edema, mild pleural effusion, and mediastinal lymphadenopathies. He received cefepime and levofloxacin for pneumonia and diuretics for heart failure. Amiodarone was stopped due to hyperthyroidism, QT prolongation, and atrial arrhythmias. He was treated with digoxin and nadolol for heart rate control.

During an echocardiogram, it was found that the left ventricle was moderately dilated with a severe reduction in ejection fraction (EF 19%) due to diffuse hypokinesis. The left atrium was moderately dilated, and the right ventricle’s longitudinal contraction was reduced. The aortic valve had three cusps, and one was suspected to be perforated, leading to severe eccentric aortic regurgitation towards the interventricular septum. A transesophageal echocardiogram confirmed the suspicion of aortic leaflet perforation, and an iso-hypoechogenic sleeve was found surrounding the aortic root and ascending segment of the aorta.

Blood cultures were drawn but were always negative; urinary antigens for Legionella pneumophila and Streptococcus pneumoniae, as well as the urine culture, were also negative. As for the possible aortic periarteritis, syphilis was excluded.

According to infectious disease specialists, as the symptoms at presentation were consistent with possible subacute endocarditis, empiric IV antibiotic treatment was initiated.

Cardiac surgery was scheduled for symptomatic valvular lesions. Vancomycin and gentamicin were given to cover possible bacteraemia during surgery. Bronchoalveolar washing before surgery showed Enterobacter cloacae sensitive to gentamicin, already in therapy.

Meanwhile, a new total-body contrast CT scan was performed to search for embolizations and define the periaortic sleeve.

The CT indicated large vessel vasculitis involving the aorta from its origin to the first descending part and the infrarenal abdominal aorta to the iliac and femoral arteries, without significant stenosis. The celiac tripod and epiaortic branches had non- critical stenosis. Aortic grafts were normal, but both coronary artery origins were involved by the same sleeve.

Coronary angiography was not deemed necessary due to suspected vasculitis and patent grafts on coronary CT. Heart catheterisation showed normal pressures and resistances, with a wedge pressure of 5 mmHg and a cardiac index of 2.72 L/min/m^2^.

A rheumatologist ordered additional labs: ESR was slightly elevated at 29, and CRP was 11.9; neutrophils were somewhat high, and there was slight proteinuria. C 3 and C 4 were normal. ANA, anti-ds-DNA, ENA, and ANCA were negative. Total IgG was slightly elevated at 1879 mg/mL, with a significant rise in IgG 4 subclass at 15.12 g/L.

A total-body PET scan showed diffuse FDG uptake in reticuloendothelial tissue, significant activity in the aortic walls and deep lymph nodes above and below the diaphragm; moreover, it also showed significant activity in the scrotum and rectal walls. Given the patient’s critical cardiac status and the surgical complexity of accessing the FDG-positive lymph nodes, the rectal mucosa was selected as a minimally invasive site to confirm systemic involvement.

Deep lymph nodes with a negative peripheral immune phenotype were monitored for follow-up; biopsy was delayed due to their deep location and unstable cardiac condition.

**Figure 1:**
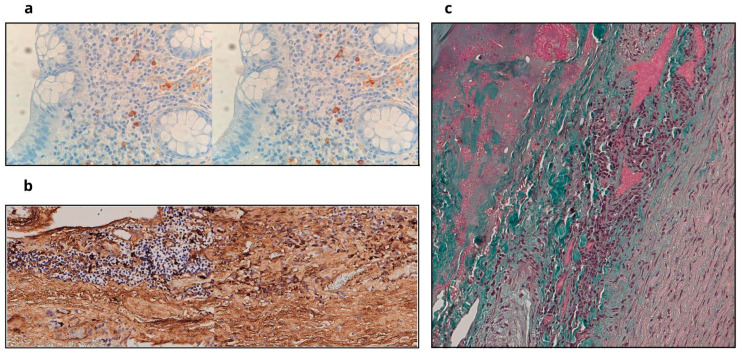
Histopathological and immunohistochemical findings supporting the diagnosis of IgG4-related disease (IgG4-RD). (**a**) Rectal submucosal biopsy (High Power Field—HPF): Immunohistochemistry analysis showing scattered IgG4-positive plasma cells (left) and IgG-positive cells (right), with a ratio < 30%. (**b**) Aortic wall biopsy (HPF), Trichrome staining: Dense inflammatory infiltrates are visible, along with characteristic areas of storiform fibrosis (stained in blue/collagen). (**c**) Aortic wall immunohistochemistry (HPF): Prominent infiltration of IgG-positive plasma cells (top) and strong, diffuse IgG4-positive staining (bottom), showing an IgG4+: IgG+ ratio of 80%.

A rectal biopsy was performed. The small specimen revealed IgG- and IgG4-positive plasma cells in perivascular spaces (Figure 1a), with a ratio of 30%, below diagnostic thresholds, and no storiform fibrosis or obliterative phlebitis due to its limited size.

In September, open-heart surgery replaced the aortic valve with an Inspiris 23 bioprosthesis. Thickened aortic walls and retracted non-coronary cusps were observed, with no perforation or signs of endocarditis. Biopsies of the aortic leaflets and wall were taken.

Histological examination of the aortic wall revealed a dense lymphoplasmacytic infiltrate with a high proportion of IgG4-positive plasma cells (>50 per HPF) and characteristic storiform fibrosis, confirming the diagnosis of IgG4-related disease (Figure 1b,c). These findings align with the international consensus diagnostic criteria for IgG4-related disease [5].

Post-surgery, the patient underwent rehab and was referred for rheumatologic follow-up. Initial treatment included steroids at 1 mg/kg/day and mycophenolate 600 mg BID as a steroid-sparing agent.

## 4. Discussion

As clearly illustrated by the case described, autoimmune processes can cause valvular heart disease through mechanisms entirely different from calcification or infection. In our patient, what appeared on echocardiography to be a cusp perforation—a hallmark of infective endocarditis—was revealed during surgery to be a significant retraction of the non-coronary cusp. This anatomical distortion resulted from dense fibrosis rather than tissue destruction. From an immunopathological perspective, IgG4 is currently regarded as a marker of the underlying inflammatory process rather than the primary pathogenic driver. The process is characterized by a complex interplay where B cells and plasmablasts act as antigen-presenting cells to CD4+ cytotoxic T cells (CTLs) [6]. These CTLs release inflammatory mediators that recruit macrophages and fibroblasts, leading to the pathognomonic storiform fibrosis observed in the surgical biopsy of the aortic leaflets [5,7]. This underlines that valvular regurgitation can be a secondary manifestation of a primary systemic fibro-inflammatory “sleeve” affecting the aortic root [8]. Crucially, as seen in our patient, valvular involvement in IgG4-RD should not be viewed as an isolated finding, as it is frequently associated with synchronous or metachronous lesions of the aortic wall, reflecting the multi-organ nature of the disease.

While IgG4-RD is a rare condition, its distribution is uneven. Evidence indicates it is more common in Eastern populations, a trend likely related to the presence of specific HLA class II antigens more frequently found in these groups [9]. Despite this, the disease is increasingly recognised globally, although often with a considerable delay in diagnosis. The case of this 40-year-old patient is especially noteworthy because the disease usually affects males aged between 50 and 70 years [3].

Additionally, clinicians should not be misled by a history of localised autoimmune conditions; in this case, the previous diagnosis of discoid lupus—treated with hydroxychloroquine—did not satisfy the American College of Rheumatology (ACR) criteria for systemic lupus erythematosus (SLE) [10]. Nevertheless, it indicated an underlying immune dysregulation.

Cardiac involvement in IgG4-RD is highly heterogeneous. Besides large vessel vasculitis involving the aorta from its origin to the iliac arteries, the disease can infiltrate the coronary arteries in three distinct patterns: diffuse wall thickening from the adventitia (observed in 92% of cases), stenotic lesions involving the media (65%), and aneurysmal dilation (42%) [11]. The “iso-hypoechogenic sleeve” identified by CT and PET in our patient involved the very origins of the coronary arteries, including the LAD and LCx, explaining the aggressive and relapsing nature of his coronary artery disease (CAD) over a decade. Even if this aggressive and relapsing CAD and the “sleeve” appearance on CT could strongly suggest a systemic vasculitis such as Takayasu’s arteritis, polyarteritis nodosa, or even a Kawasaki-like syndrome secondary to SARS-CoV-2 infection, it was definitely excluded by the aortic leaflet histological analysis [12].

Interestingly, IgG4-RD can also cause left ventricular ejection fraction depression through small vessel vasculitis with lymphocyte infiltration; however, unlike other manifestations, this specific process is often not responsive to steroids [13]. This emphasizes the need for comprehensive imaging; in this patient, the absence of heart uptake on the PET scan ruled out small vessel involvement, thereby concentrating the pathology on the major vascular structures.

The diagnostic confirmation of IgG4-RD disease relies on the 2019 ACR/EULAR classification criteria, which use a weighted scoring system designed to distinguish this syndrome from its many mimics [14]. This structured process follows a three-step approach. First, the patient must meet entry criteria through characteristic clinical or radiological involvement of a typical organ. Second, they must not meet any of the specified exclusion criteria, which rule out infectious, malignant, or other autoimmune conditions. In this context, chronic rheumatic heart disease was explicitly excluded based on the clinical history—not meeting the modified Jones criteria—and the absence of Aschoff bodies upon histological review [15]. The presence of pathognomonic storiform fibrosis (Figure 1b) and dense IgG4-positive infiltration (Figure 1c) clearly distinguishes this case from rheumatic valvulitis. Third, patients are assessed across several inclusion domains, including histopathology, serology, and imaging. A cumulative score of 20 or more is required for classification, with a high specificity of up to 99.2%.

In our patient’s case, the total score was 39 points, significantly above the required threshold. This definitive classification was primarily influenced by the histopathology domain, where the presence of a dense lymphocytic infiltrate and storiform fibrosis contributed 13 points, and by the serology domain, where serum IgG4 levels exceeding five times the upper limit of normal added another 11 points. Moreover, the immunostaining of the aortic wall—showing an IgG4+: IgG+ ratio of 80% with over 50 IgG4+ cells per high-power field—provided the maximum weight for that specific criterion. It is noteworthy that the decision to perform a rectal biopsy was guided by the total-body PET scan, which showed significant FDG uptake in the rectal wall; this served as a minimally invasive site to confirm systemic involvement (Figure 1a) in a patient with cardiac instability. This biopsy permitted an earlier assessment yielding a score of 25 points, which would have already been sufficient for diagnosis. This illustrates the utility of the scoring system even when primary organ samples are initially unavailable.

Finally, in high-risk cases of suspected valvulitis, risk stratification and infection prevention remain paramount [16]. The use of preventive measures, such as gentamicin-impregnated collagen sponges, should be considered to minimize post-operative complications, even when an immune-mediated etiology is suspected [17].

## 5. Conclusions

Management of heart involvement in IgG4-RD requires close vigilance and a multidisciplinary approach. Cardiologists must look beyond conventional risk factors—such as smoking and dyslipidaemia present in this case—when coronary disease progresses rapidly or recurs despite optimal revascularisation. In this regard, adopting standardised ‘red flags’ and ‘clinical gateways’ for rare conditions, as advocated in the Argo Delphi consensus statement by Limongelli et al. (2025), can significantly assist cardiologists in navigating the complex diagnostic journey of IgG4-RD and other uncommon systemic disorders [18].

Ultimately, the successful management of such a rare and systemic condition requires a multidisciplinary approach. The ‘tips for cardiologists’ derived from this case emphasise that no single specialist can manage IgG4-RD or other rare multisystemic diseases in isolation; it requires a coordinated network involving rheumatologists, radiologists, clinical pathologists, and surgeons to avoid diagnostic delays and optimise treatment. Moreover, long-term management involves collaboration with rheumatologists to balance corticosteroid therapy with steroid-sparing agents, such as mycophenolate mofetil. This is vital because relapses frequently occur during steroid tapering. Ultimately, referring these patients to specialised centres is crucial to ensure that the “masked” manifestations of IgG4-RD are correctly identified and treated before irreversible organ damage occurs.

## Data Availability

The original contributions presented in this study are included in the article. Further inquiries can be directed to the corresponding author.
